# Molecular resistance mechanisms to newly approved antibiotics (2017–2025) in WHO priority pathogens

**DOI:** 10.3389/fmicb.2025.1719798

**Published:** 2026-01-13

**Authors:** M. Sartori, S. Toppo, E. Lavezzo

**Affiliations:** Department of Molecular Medicine, University of Padova, Padova, Italy

**Keywords:** antimicrobial resistance (AMR), molecular diagnostics, novel antibiotics, resistance mechanisms, WHO priority pathogens

## Abstract

The relentless rise of antimicrobial resistance (AMR) poses a critical threat to global public health, rendering once-effective therapies obsolete. In response, several novel antibiotics have been developed in recent years. This review systematically summarizes the molecular resistance mechanisms that World Health Organization (WHO) priority bacterial pathogens have already deployed against the 15 new antibiotics approved between 2017 and 2025, including β-lactam/β-lactamase inhibitors (cefiderocol, ceftazidime-avibactam, meropenem-vaborbactam), tetracycline derivatives (eravacycline, omadacycline), a pleuromutilin (lefamulin), an aminoglycoside (plazomicin), and a fluoroquinolone (delafloxacin). We detail how pathogens utilize four primary strategies to overcome these last-line agents: enzymatic inactivation (e.g., by KPC, NDM, OXA-48, and Tet(X) variants), efflux pump overexpression (e.g., AdeABC, AcrAB-TolC, MexAB-OprM), modifications of target sites (e.g., PBP3, RpoB, ribosomal proteins/L3, and QRDR mutations), and reduced membrane permeability. Evidence consistently demonstrates that resistance emerges rapidly, often through pre-existing genetic pathways repurposed against the new chemical structures. This analysis underscores the paradoxical reality of antimicrobial development: the introduction of new therapies simultaneously selects for and elucidates new resistance mechanisms. Preserving the efficacy of these essential drugs thus necessitates a multifaceted, globally coordinated “One Health” strategy. Finally, we discuss how the growing complexity of AMR mechanisms is driving the need for advanced diagnostic tools, exploring the pivotal role of bioinformatics and artificial intelligence in predicting resistance and closing knowledge gaps.

## Introduction

The global rise of antimicrobial resistance (AMR) poses a major threat to public health, diminishing the effectiveness of current antibiotics and making it increasingly difficult to treat both common and severe infections (Naghavi et al., 2024).

In response to this challenge, the World Health Organization (WHO) developed a priority list of pathogenic bacteria, classifying them as critical, high or medium, based on factors such as transmissibility, mortality, prevalence, availability of effective treatments, and potential for future spread (WHO, 2024) (Table 1). The aim is to highlight which pathogens pose an emerging threat in terms of AMR and to guide the setting of priorities for the development of new therapeutic strategies.

**Table 1:** List of WHO bacterial priority pathogens of public health importance, 2024 update.

Critical group	High group	Medium group
*Acinetobacter baumannii* carbapenem-resistant	*Salmonella Typhi* fluoroquinolone-resistant	Group A Streptococci macrolide-resistant
Enterobacterales third-generation cephalosporin-resistant	*Shigella* spp. fluoroquinolone-resistant	*Streptococcus pneumoniae* macrolide-resistant
Enterobacterales carbapenem-resistant	*Enterococcus faecium* vancomycin-resistant	*Haemophilus influenzae* ampicillin-resistant
	*Pseudomonas aeruginosa* carbapenem-resistant	Group B Streptococci penicillin-resistant
	Non-typhoidal *Salmonella* fluoroquinolone-resistant	
	*Neisseria gonorrhoeae* third-generation cephalosporin, and/or fluoroquinolone-resistant	
	*Staphylococcus aureus* methicillin-resistant	

Meanwhile, between 2017 and 2025, several new antibiotics were approved for systemic use by the Food and Drug Administration (FDA) and the European Medicines Agency (EMA) to address infections caused by drug-resistant organisms (Table 2). However, resistance to many of these agents has already emerged, highlighting both the remarkable adaptability of bacterial pathogens and the complexity of AMR dynamics.

**Table 2:** Antimicrobial drugs approved by FDA and/or EMA since 2017.

Entry	Drug name	Approval year	Drug class *(subclass)*	Main use
1.	Aztreonam and avibactam	2024 EMA	β-lactams and BLICs *Monobactam and non-β-lactam β-lactamase inhibitor*	cUTIs, cIAIs, HABP, VABP, and Gram-negative blood stream infections with limited treatment options
2.	Cefepime and enmetazobactam	2024 FDA	β-lactams and BLICs *Cephalosporin and β-lactamase inhibitor*	HABP and cUTIs
3.	Ceftobiprole	2024 FDA	β-lactams and BLICs *Cephalosporin*	ABSSSI, CABP and SAB
4.	Sulbactam and durlobactam	2023 FDA	β-lactams and BLICs *β-lactamase inhibitors*	*A. baumannii–calcoaceticus* complex HABP and VABP in adult patients aged
5.	Cefiderocol	2019 FDA 2020 EMA	β-lactams and BLICs *Cephalosporin*	cUTIs, HABP, VABP, and other serious Gram-negative infections with limited treatment options
6.	Imipenem, cilastatin, and relebactam	2019 FDA 2020 EMA	β-lactams and BLICs *Carbapenem, renal dehydropeptidase inhibitor, and β-lactamase inhibitor*	cUTIs, cIAIs, and HABP/VABP
7.	Meropenem and vaborbactam	2017 FDA 2018 EMA	β-lactams and BLICs *Carbapenem and β-lactamase inhibitor*	cUTIs, cIAIs, HAP/VAP, and bacteremia or other infections caused by CRE with limited treatment options
8.	Pretomanid	2019 FDA 2020 EMA	Nitroimidazole	Pulmonary TB (MDR-TB and RR-TB)
9.	Lefamulin	2019 FDA 2020 EMA	Pleuromutilin	CABP
10.	Omadacycline	2018 FDA	Tetracycline	ABSSSI, CABP
11.	Eravacycline	2018 FDA 2018 EMA	Tetracycline	cIAIs
12.	Plazomicin	2018 FDA	Aminoglycoside	cUTIs
13.	Rifamycin	2018 FDA	Rifamycin	Travelers’ diarrhea caused by non-invasive strains of *Escherichia coli*
14.	Delafloxacin	2017 FDA 2019 EMA	Fluoroquinolone	ABSSSIs, CABP
15.	Gepotidacin	2025 FDA	Triazaacenaphthylenes	uUTI

All abbreviations used in this table are defined in Glossary section. The grey shading was used to separate the different drug classes. The fourth column “drug class (subclass)” provides this info, and all consecutive drugs belonging to the same class were colored together (either grey or white).

This review aims to systematically summarize the resistance mechanisms reported for WHO-listed priority bacterial pathogens against the new antibiotics approved for systemic use between 2017 and 2025. We limited our analysis to antibiotics first approved by either the FDA or the EMA. This decision is supported by evidence that new antibiotics originating from companies in the US or Europe historically achieved significantly greater geographic availability than those first approved elsewhere (Kållberg et al., 2018). The marked variability in global diffusion further indicates that many agents, particularly those originating outside the US/Europe, never obtain wide international registration. For example, antibiotics first marketed in Japan have rarely expanded beyond Asia (Kållberg et al., 2018). Because our aim is to review resistance mechanisms with likely global relevance based on clinical use, focusing on FDA/EMA-approved antibiotics ensures that selected molecules have had greater opportunity for dissemination and real-world use across multiple regions, thereby maximizing the relevance and generalizability of the resistance data. We acknowledge, however, that this choice excludes agents approved only by other regulatory agencies, which may remain important in local contexts.

FDA approvals were identified by consulting the official approval documentation (PDF drug labels) for each individual antibiotic. EMA approvals were retrieved through manual searches of the EMA medicines database for antibacterial agents within the relevant timeframe.^1^ To ensure completeness, these data were cross-checked against the WHO antibacterial pipeline reports (2017–2023) and the review *“Approved antibacterial drugs in the last 10 years: from the bench to the clinic”* (García-Castro et al., 2023). Antibiotics first approved exclusively by regulatory agencies outside the FDA or EMA, such as lascufloxacin, levonadifloxacin, or contezolid, were not included, as well as molecules withdrawn from the market, new formulations of older agents, and antibiotics approved prior to 2017. No additional criteria based on indication type (e.g., orphan or niche use) were applied. The complete list of antibiotics considered in this review is reported in Table 2. To identify all published molecular resistance mechanisms associated with these antibiotics, we conducted a structured literature search in PubMed. For each drug, we queried *“antibiotic resistance AND <drug name>”* and included studies reporting experimentally supported molecular resistance determinants in WHO priority pathogens.

By mapping the resistance landscape in relation to newly approved drugs, our work provides a comprehensive overview of the challenges threatening their effectiveness and supports ongoing efforts in antimicrobial stewardship, surveillance, and drug development. Furthermore, we explore how this detailed mechanistic knowledge underscores the need for, and informs the development of, next-generation diagnostic tools, including those leveraging bioinformatics and artificial intelligence.

The review is organized by antibiotic class. For each class, we first provide a concise overview of its mechanism of action and general resistance mechanisms, followed by dedicated sections for each specific drug that comprehensively detail the molecular resistance mechanisms identified in WHO priority pathogens. The order of presentation follows a logical progression, beginning with novel β-lactam combinations and agents (including β-lactam/β-lactamase inhibitors and the siderophore cephalosporin cefiderocol), followed by newer tetracyclines, pleuromutilins, aminoglycosides, fluoroquinolones, and the novel triazaacenaphthylene class. The scope also extends to the nitroimidazole pretomanid for tuberculosis. Finally, we summarize these findings to discuss the overarching implications for public health and future antibiotic development, concluding with an analysis of the role of advanced computational methods in the ongoing battle against AMR.

Because the antibiotics included in this review were all approved between 2017 and 2025, quantitative data on the prevalence of specific resistance mechanisms remain extremely limited. For most agents, published evidence consists of surveillance panels with very small sample sizes, isolated clinical case reports, in-vitro studies, or experimental evolution models. Therefore, the frequency of individual molecular mechanisms (e.g., specific PBP mutations, β-lactamase variants, or efflux-related changes) cannot yet be reliably estimated in real-world clinical populations. For this reason, the present review focuses on documenting the molecular and genetic resistance mechanisms described to date, while explicitly acknowledging that their current prevalence and clinical impact cannot be robustly quantified for most newly approved antibiotics. As post-marketing use expands and larger surveillance datasets accumulate, more accurate prevalence estimates will likely become available.

## β-Lactams

*Overview.* β-lactams are a broad class of antibiotics characterized by the presence of a β-lactam ring in their chemical structure. This group includes penicillins, cephalosporins, carbapenems, and monobactams, which together represent some of the most widely used and clinically important antimicrobials (see Table 3).

**Table 3:** β-lactams mechanisms of action and resistance.

β-lactams	
Mechanism of action	Resistance mechanisms
Inhibit PBPs/transpeptidase activity, blocking peptidoglycan cross-linking and cell wall synthesis	Reduced permeability (intrinsic outer membrane barrier or porin loss)
Cause cell lysis	β-lactamase-mediated enzymatic inactivation (counteracted by β-lactamase inhibitors)
	Efflux pump upregulation
	PBP mutations or acquisition of low-affinity PBPs
	β-lactamase-mediated inactivation

*Mechanism of action*. β-lactam antibiotics selectively and irreversibly inhibit the transpeptidase activity of penicillin-binding proteins (PBPs), enzymes responsible for the final step of peptidoglycan cross-linking in bacterial cell wall synthesis. This transpeptidase activity catalyses the formation of cross-links (such as pentaglycine bridges) between peptidoglycan polymers, a process essential for building the three-dimensional bacterial cell wall and maintaining cell morphology. PBPs are a family of enzymes whose roles vary among bacterial species and are not yet fully understood. Owing to their structural similarity to the natural PBP substrate (the D-Ala-D-Ala terminus of the peptidoglycan peptide stem), β-lactams acylate the active site of PBPs and block cell wall synthesis, ultimately leading to bacterial death via cell lysis.

*General resistance mechanism*. Resistance to β-lactams can be either intrinsic or acquired. Intrinsic resistance in Gram-negative bacteria is often due to the outer membrane acting as a permeability barrier. Acquired resistance arises through multiple mechanisms, including: (i) reduced permeability due to the variation in the size and number of porins; (ii) increased drug efflux mediated by efflux pumps; (iii) modification or overproduction of the target, such as the acquisition of alternative, low-affinity PBPs or mutations in existing PBPs that reduce drug binding; and (iv) enzymatic inactivation by β-lactamases, which hydrolyse the β-lactam ring, inactivating the drug. To counteract the latter mechanism, β-lactams are frequently administered in combination with β-lactamase inhibitors, which act as “suicide substrates” by irreversibly inactivating bacterial β-lactamases.

### Aztreonam-avibactam

*Overview.* Aztreonam-avibactam is a combination antibiotic used to address infections caused by multidrug-resistant Gram-negative bacteria. It pairs aztreonam, a monobactam β-lactam, with avibactam, a β-lactamase inhibitor. Aztreonam is intrinsically stable to hydrolysis by metallo-β-lactamases (MBLs), but its clinical utility has been limited by susceptibility to extended-spectrum β-lactamases (ESBLs) and AmpC enzymes. Avibactam complements aztreonam by inhibiting class A, class C, and some class D β-lactamases, thereby restoring activity against bacterial strains that co-produce MBLs and other β-lactamases (Ehmann et al., 2013). This combination has shown particular relevance for the treatment of carbapenem-resistant *Enterobacterales* (CRE), including *Klebsiella pneumoniae* (*K. pneumoniae*) and *Escherichia coli* (*E. coli*), as well as other resistant Gram-negative pathogens (Chen et al., 2022).

*Clinical use.* Aztreonam–avibactam is indicated for complicated urinary tract infections (cUTIs), complicated intra-abdominal infections (cIAIs), hospital-acquired bacterial pneumonia (HABP) [including ventilator-associated bacterial pneumonia (VABP)], and bloodstream infections caused by multidrug-resistant organisms, representing an important option where therapeutic alternatives are extremely limited.

*Resistance mechanisms.* All four of the classical β-lactam resistance strategies have been reported in relation to aztreonam–avibactam.

- *Reduced outer membrane permeability.* Mutations affecting porins are a recurrent mechanism in *Enterobacterales* and *Pseudomonas aeruginosa* (*P. aeruginosa*). In *E. coli*, alterations in OmpC and OmpF reduce drug uptake, while in *K. pneumoniae* resistance has been linked to the loss or modification of OmpK35 and OmpK36, particularly in isolates that co-produce *K. pneumoniae* carbapenemase (KPC) or CMY-type β-lactamases (Yu et al., 2022; Tellapragada et al., 2024). In *P. aeruginosa*, decreased susceptibility has been associated with the loss of OprD, a channel that normally facilitates the uptake of β-lactams and other small molecules (González-Pinto et al., 2024).
- *Increased efflux pump expression*. Overexpression of the AcrAB-TolC system in *E. coli* and *K. pneumoniae* contributes to the active extrusion of aztreonam, especially when combined with reduced porin-mediated uptake (Yu et al., 2022; Tellapragada et al., 2024). In *P. aeruginosa*, the MexAB-OprM efflux system plays a similar role, exporting β-lactams and other antibiotics and thereby reinforcing multidrug resistance (González-Pinto et al., 2024).
- *Modification of target sites*. Alterations in penicillin-binding protein 3 (PBP3), the main target of aztreonam, have been documented in *E. coli*. A characteristic four–amino acid insertion (Tyr-Arg-Ile-Asn or Tyr-Arg-Ile-Lys) reduces binding affinity for aztreonam, impairing its bactericidal activity (Tellapragada et al., 2024; Le Terrier et al., 2024; Helsens et al., 2024; Long et al., 2025b; Bakthavatchalam et al., 2023; Ma and Zong, 2022; Le Terrier et al., 2023; Mendes et al., 2021). This mechanism is often potentiated by concurrent β-lactamase production (Ma et al., 2020).
- *Enzymatic inactivation*. β-lactamases production remains the most widespread threat to aztreonam–avibactam.
- In *E. coli*, expression of the AmpC variant CMY-42, as well as SHV-12, has been associated with reduced susceptibility, the latter due to structural changes preventing avibactam inhibition (Helsens et al., 2024; Long et al., 2025b; Le Terrier et al., 2023; Mendes et al., 2021; Wu et al., 2023; Mushtaq et al., 2021).
- In *K. pneumoniae*, resistance has been observed in strains with high-level or mutated KPC expression, a subclass of β-lactamases that can hydrolyse carbapenems. Overproduction of KPC can saturate avibactam’s inhibitory capacity (Yu et al., 2022; Xiang et al., 2025), while mutations such as Trp105Arg in KPC-2 (defining the KPC-21 variant) further reduce inhibitor binding (Niu et al., 2020; Ma et al., 2022). Other β-lactamases, including CMY-16, have also been implicated (Niu et al., 2020).
- In *P. aeruginosa*, resistance has been linked to New Delhi Metallo-β-lactamase 1 (NDM-1) expression (González-Pinto et al., 2024).
- *Putative additional mechanisms.* Beyond classical pathways, emerging evidence points to novel resistance determinants. For example, a mutation in the *P. aeruginosa PA4292* gene leads to increased pyocyanin production, which may indirectly promote β-lactam resistance, highlighting the possibility of yet-uncharacterized resistance pathways (Zhao et al., 2022).

### Cefepime-enmetazobactam

*Overview.* Cefepime–enmetazobactam combines cefepime, a fourth-generation cephalosporin, with enmetazobactam, a novel β-lactamase inhibitor structurally related to tazobactam but with improved activity against ESBLs. The addition of enmetazobactam restores cefepime activity against ESBL-producing *Enterobacterales*, expanding treatment options for multidrug-resistant (MDR) Gram-negative infections (Bhowmick et al., 2025).

*Clinical use.* Cefepime–enmetazobactam is approved for the treatment of HABP, cUTIs, and cIAIs (Bhowmick et al., 2025).

*Resistance mechanisms.* To date, specific resistance mechanisms to cefepime-enmetazobactam have not been fully elucidated. Available data largely describe decreased efficacy rather than well-defined molecular pathways. The main determinant of reduced activity appears to be the presence of carbapenemase-producing strains.

- *KPC and metallo-β-lactamases. Enterobacterales* carrying KPC or MBLs show markedly lower susceptibility, suggesting that enmetazobactam does not efficiently inhibit these enzymes (Vázquez-Ucha et al., 2022; Lee et al., 2021; Blanco-Martín et al., 2024; Morrissey et al., 2024).
- *OXA-48 carbapenemases.* Evidence is conflicting. Some European studies report retained susceptibility in OXA-48-producing *Enterobacterales*, whereas later reports demonstrated resistance in similar strains (Vázquez-Ucha et al., 2022; Morrissey et al., 2024).
- *Putative additional mechanisms.* Unlike other recently approved β-lactam/β-lactamase inhibitor combinations, no consistent molecular resistance mechanisms (e.g., porin loss, efflux, PBP modifications) have yet been characterized for cefepime–enmetazobactam. Further surveillance and mechanistic studies will be essential to determine whether resistance will emerge through known β-lactam pathways or novel mechanisms.

### Ceftobiprole

*Overview.* Ceftobiprole is a fifth-generation cephalosporin effective against both Gram-positive and Gram-negative pathogens and is particularly valuable for its efficacy against methicillin-resistant *Staphylococcus aureus* (MRSA) (Holland et al., 2023).

*Clinical use.* It is approved for the treatment of acute bacterial skin and skin structure infections (ABSSSI), community-acquired bacterial pneumonia (CABP), and *S. aureus* bacteraemia (SAB).

*Resistance mechanisms.* Although ceftobiprole is generally effective, resistant isolates have already been reported, particularly in MRSA but also in *Enterococcus faecalis* (*E. faecalis*) and *Corynebacterium jeikeium* (*C. jeikeium*) (Lazzaro et al., 2021; Lavollay et al., 2024; Conti et al., 2024). The main mechanism of resistance involves mutations in PBPs:

- *PBP2a mutations*. Resistance in MRSA is often associated with alterations in PBP2a. African isolates have shown a triple mutation (N146K–N204K–G246E), which confers high-level resistance to both ceftaroline and ceftobiprole (Schaumburg et al., 2016). Italian isolates, by contrast, carry a distinct set of mutations (N146K–N204K–T235I–E239K) in the non-penicillin-binding domain of PBP2a (Morroni et al., 2018; Bongiorno et al., 2019; Mlynarczyk-Bonikowska et al., 2022).
- *PBP4 mutations*. Both missense and promoter mutations in PBP4 have been implicated in enhancing resistance to ceftobiprole and other β-lactams in *E. faecalis* and *S. aureus* (Lazzaro et al., 2021; Greninger et al., 2016; Hamilton et al., 2017; Alexander et al., 2018).
- *Putative additional mechanisms.* Additional mutations have been described in *mecA*, *mecC, guaA*, *guaB*, *relA, rpoA,* and *oatA*, as well as alterations in the GdpP protein. While their precise role is still being elucidated, these changes appear to contribute to the complex resistance phenotype (Morroni et al., 2018; Bongiorno et al., 2019; Mlynarczyk-Bonikowska et al., 2022; Zhu et al., 2022).

### Sulbactam-durlobactam

*Overview.* Sulbactam-durlobactam is a novel antimicrobial combination developed to treat infections caused by *Acinetobacter baumannii* (*A. baumannii*), particularly carbapenem-resistant strains. Sulbactam is a β-lactam antibiotic with intrinsic activity against *A. baumannii*, while durlobactam is a serine β-lactamase inhibitor that restores sulbactam’s activity by inhibiting class A, C, and some class D β-lactamases (McLeod et al., 2024).

*Clinical use.* The combination is approved for the treatment of HABP and VABP caused by the *A. baumannii–calcoaceticus* complex (ABC) in patients aged 18 years and older (McLeod et al., 2024).

*Resistance mechanisms*. Multiple resistance pathways have already been described in *A. baumannii*:

- *Efflux pump overexpression*. Mutations in the *adeIJK* operon, particularly in *adeJ* (encoding the membrane fusion protein of the AdeIJK efflux system), increase the efflux of durlobactam, thereby reducing its efficacy (Principe et al., 2022; Moussa et al., 2023).
- *Target site modifications.* Substitutions in PBP3, especially A515V and T526S near the active site, have been linked to reduced susceptibility (Iovleva et al., 2024).
- *Enzymatic inactivation*. The presence of NDM-1 has been associated with markedly elevated MICs (>32 mg/L) in a subset of resistant isolates (Principe et al., 2022).
- *Putative additional mechanisms*. Additional substitutions in PBP3, such as Q488K and Y258H, have been reported, although their role in resistance remains less clear (Principe et al., 2022; Moussa et al., 2023). Alterations in other PBPs, including PBP1a, PBP1b, and PBP2, may also contribute (Iovleva et al., 2024; Findlay et al., 2022).

### Cefiderocol

*Overview.* Cefiderocol is a siderophore cephalosporin designed for the treatment of complicated infections caused by MDR Gram-negative bacteria. It acts as a “Trojan horse” antibiotic, chelating iron and exploiting bacterial iron uptake systems for cell entry. Its high affinity for PBP3, together with its ability to bypass efflux pumps and resist hydrolysis by most β-lactamases (including MBLs), underpins its broad-spectrum activity. Cefiderocol shows potent *in vitro* efficacy against carbapenem-resistant pathogens such as *K. pneumoniae*, *E. coli*, *P. aeruginosa*, *A. baumannii*, and *Stenotrophomonas maltophilia*. Notably, it retains activity against strains producing both serine β-lactamases (e.g., KPC, OXA-48) and MBLs (e.g., NDM, VIM, IMP), making it a valuable last-line therapeutic option (Russo and Serapide, 2025).

*Clinical use.* Cefiderocol is approved for the treatment of cUTI, HABP, VABP, and other serious infections due to aerobic Gram-negative bacteria when alternative options are limited (Russo and Serapide, 2025).

*Resistance mechanisms*. Resistance to cefiderocol involves multiple adaptive strategies, reflecting its dual reliance on iron transport systems and β-lactam activity.

*Alterations in iron transport systems*. Since cefiderocol relies on TonB-dependent siderophore receptors for entry, mutations or truncations in these receptors may reduce drug uptake.

- *A. baumannii*: Mutations in *pirA* (premature stop codons, insertions) and *piuA* (truncated proteins) reduce receptor function; alterations in the TonB–ExbB–ExbD system further impair entry (He et al., 2022; Alteri et al., 2024; Huang et al., 2024; Strateva and Peykov, 2024; Malik et al., 2020; Tantry et al., 2024; Shields et al., 2024; Findlay et al., 2024a; Tiseo et al., 2023; Asrat et al., 2023; Yamano et al., 2022).
- *E. coli*: Mutations in *cirA* are linked to resistant phenotypes (Barker et al., 2024; Simner et al., 2023; Kocer et al., 2023; Poirel et al., 2022; Wang et al., 2022).
- *K. pneumoniae*: Inactivation or truncation of receptors such as *fiu, fepA, fhuA, iutA* and especially *cirA* are associated with reduced susceptibility (Galani et al., 2025; Long et al., 2025a; Polani et al., 2025; Deroche et al., 2025; Yang et al., 2024; Hong et al., 2024; Freiberg et al., 2024; Arcari et al., 2023; Padovani et al., 2023; Coppi et al., 2022; Lan et al., 2022; McElheny et al., 2021).
- *P. aeruginosa*: Mutations affect multiple outer membrane receptors (*cirA*, *fptA*, *piuA*, *pirA*) and transport components (*piuC/D, pirS/C*), including complete deletion of *piuC* in some isolates (González-Pinto et al., 2024; Oliver et al., 2025; Viñes et al., 2025; Egge et al., 2024; Gomis-Font et al., 2024; Gomis-Font et al., 2023).

*Reduced outer membrane permeability*. Mutations in porins, regulatory systems, and energy maintenance pathways decrease intracellular cefiderocol levels.

- *A. baumannii*: Mutations in the BaeS–BaeR two-component system (stress response) contribute to resistance (Liu et al., 2023).
- *K. pneumoniae*: Alterations in OmpK35/OmpK36 porins and EnvZ/OmpR signaling reduce permeability (Castillo-Polo et al., 2023; Di Pilato et al., 2024; Amadesi et al., 2024; Boattini et al., 2024; Moon and Huang, 2023).
- *E. coli*: Mutations in *tolR* (Tol–Pal system) impair membrane stability and drug activity (Castillo-Polo et al., 2024).

*Efflux pumps overexpression.* In *P. aeruginosa*, activation of the ParRS and CpxRS systems induces efflux mechanisms, notably overexpression of the MexAB–OprM pump, which expels cefiderocol. Loss of OprD porin further reduces entry (González-Pinto et al., 2024; Sastre-Femenia et al., 2025).

*Target site modifications*. Mutations in PBP3 have been identified in resistant isolates of *A. baumannii* (Malik et al., 2020; Stracquadanio et al., 2024; Kriz et al., 2024), E. coli (Barker et al., 2024; Simner et al., 2023; Wang et al., 2022; Haidar et al., 2024; Rodríguez-Villodres et al., 2024), and P. aeruginosa (Oliver et al., 2025; Gomis-Font et al., 2023; Kocer et al., 2024). In *K. pneumoniae,* resistance has been linked to alterations in PBP2 (Castillo-Polo et al., 2023). These mutations impair drug–target binding.

*Enzymatic inactivation.* β-lactamases and carbapenemases remain a major resistance driver:

- *A. baumannii*: class D carbapenemases (OXA-23, OXA-24, OXA-58) and metallo-β-lactamases (notably NDM variants) reduce susceptibility, often in combination with permeability defects (He et al., 2022; Huang et al., 2024; Traglia et al., 2024; Sánchez-Urtaza et al., 2023b; Sánchez-Urtaza et al., 2023a; Rodríguez-Aguirregabiria et al., 2024; Gaillot et al., 2023; Desmoulin et al., 2024).
- *E. coli*: Production of NDM enzymes (e.g., NDM-5, NDM-35) significantly raises MICs; plasmid AmpC β-lactamases (CMY-59, CMY-145) further contribute, particularly when combined with mutations in *ftsI* (Barker et al., 2024; Kocer et al., 2023; Poirel et al., 2022; Wang et al., 2022; Haidar et al., 2024; Martin et al., 2024).
- *K. pneumoniae*: Variants of KPC (KPC-245, KPC-33, KPC-31) and coexpression of NDM metallo-β-lactamases (e.g., NDM-1 and NDM-5) impair cefiderocol activity, particularly when coupled with siderophore receptor mutations (Long et al., 2025a; Long et al., 2025a; Yang et al., 2024; Hong et al., 2024; Castillo-Polo et al., 2023; Di Pilato et al., 2024; Amadesi et al., 2024; Amadesi et al., 2024; Bovo et al., 2023; Gaibani et al., 2022a; Birgy et al., 2024; Bellinzona et al., 2024; Gaibani et al., 2022b). Overexpression of SHV β-lactamases also elevates MICs (Hong et al., 2024).
- *P. aeruginosa*: Resistance involves mutations in AmpC and OXA-type enzymes (e.g., OXA-2, OXA-10, OXA-46), as well as production of VIM, NDM, and IMP carbapenemases (González-Pinto et al., 2024; Oliver et al., 2025; Findlay et al., 2024b; Stoikov et al., 2023; Benzaarate et al., 2023; Vuillemin et al., 2023). Overexpression of PER-1, often via mobile genetic elements, drives cross-resistance with ceftazidime-avibactam (Papa-Ezdra et al., 2023; Wang et al., 2023).

All the β-lactamases identified in resistant strains are reported in Supplementary Table S1.

### Imipenem-cilastatin-relebactam

*Overview.* Imipenem-cilastatin-relebactam is a fixed-dose combination of three agents designed to restore carbapenem activity against MDR Gram-negative bacteria. Imipenem is a broad-spectrum carbapenem antibiotic; cilastatin is a renal dehydropeptidase inhibitor that prevents the degradation of imipenem in the kidneys; and relebactam is a novel diazabicyclooctane β-lactamase inhibitor. This combination expands the activity of imipenem against CRE (particularly *K. pneumoniae*) and *P. aeruginosa.*

*Clinical use.* The drug is approved for the treatment of cUTI, cIAI, and HABP/VABP. Its main role is in the management of infections caused by carbapenem-resistant Gram-negative pathogens where treatment options are limited (Heo, 2021).

*Resistance mechanisms.* Resistance to imipenem–cilastatin–relebactam arises mainly from alterations in membrane permeability, efflux regulation, and the presence of β-lactamases not inhibited by relebactam.

- *K. pneumoniae*: Resistance is strongly associated with loss or combined mutation of the *ompK35* and *ompK36* porin genes, which encode the major outer membrane channels for carbapenems. These mutations restrict drug entry and, when combined with the production of carbapenemases (particularly KPC variants), lead to marked resistance (Boattini et al., 2024; Palomba et al., 2025; Gaibani et al., 2022d). Additional resistance can be mediated by other β-lactamases, including NDM, OXA-type carbapenemases, and CTX-M ESBLs (Gaibani et al., 2022d; Bedenić et al., 2023).
- *P. aeruginosa*: Mechanisms are more diverse and involve chromosomal mutations. Recent genomic studies have shown that mutations in *ftsI* (encoding PBP3, the target of imipenem), *nalD* (a negative regulator of the MexAB-OprM efflux pump), and *pvdS* (a sigma factor regulating stress and iron uptake) are enriched in resistant strains (Rapsinski et al., 2025). Together, these alterations reduce antibiotic binding, enhance efflux activity, and adapt bacterial physiology to survive drug pressure.

### Meropenem-vaborbactam

*Overview.* Meropenem–vaborbactam is a fixed-dose combination of a carbapenem antibiotic with a novel boronic acid–based β-lactamase inhibitor. Vaborbactam was specifically developed to restore meropenem activity against CRE by inhibiting class A carbapenemases (especially KPC) and, to a lesser extent, certain class C β-lactamases (AmpC). However, it has no activity against MBLs (NDM, VIM, IMP) or OXA-type carbapenemases, limiting its spectrum compared with other novel β-lactam/β-lactamase inhibitor combinations.

*Clinical use.* The combination is approved for the treatment of cUTI (including pyelonephritis), cIAI, and HAP, including VAP. It may also be used for bacteremia or other infections caused by CRE when alternative treatments are not suitable.

*Resistance mechanism.* Despite the addition of vaborbactam, resistance can arise through multiple mechanisms that impair antibiotic entry, increase drug efflux, or overwhelm the inhibitor with excessive β-lactamase activity.

*Reduced outer membrane permeability*.

- *K. pneumoniae:* Resistance is frequently mediated by mutations or loss of porins that normally facilitate carbapenem entry.
- OmpK35 inactivation: loss-of-function mutations abolish passage of hydrophilic molecules, further restricting entry (Boattini et al., 2024; Başaran and Öksüz, 2025; Yasmin et al., 2024; Bongiorno et al., 2022; Gaibani et al., 2022e; Gaibani et al., 2022c; Sun et al., 2017; Bianco et al., 2025; Dulyayangkul et al., 2020; Gaibani et al., 2020).
- OmpK36 mutations: Insertions at positions Gly134-Asp135 narrow the channel and reduce meropenem influx (Boattini et al., 2024; Başaran and Öksüz, 2025; Yasmin et al., 2024; Bongiorno et al., 2022; Gaibani et al., 2022e; Gaibani et al., 2022c; Sun et al., 2017; Bianco et al., 2025; Dulyayangkul et al., 2020; Gaibani et al., 2020; Rogers et al., 2023a; Findlay et al., 2023; Satapoomin et al., 2022; Cienfuegos-Gallet et al., 2024; Di Marcantonio et al., 2024; Rogers et al., 2023b; Hayden et al., 2020).
- OmpK37 alterations: mutations occasionally affect this alternative porin (Boattini et al., 2024; Bongiorno et al., 2022; Di Marcantonio et al., 2024).
- Additional contributors include inactivation of KvrA, a transcriptional regulator whose loss reduces OmpK35/36 expression, and loss of NlpD, a cell wall remodelling protein linked to decreased porin abundance (Başaran and Öksüz, 2025; Dulyayangkul et al., 2020).
- *P. aeruginosa*: Resistance is commonly associated with mutations or loss of *oprD*, the specific porin for carbapenem uptake (González-Pinto et al., 2024; Sastre-Femenia et al., 2025).

*Increased efflux pump activity.* Overexpression of efflux systems can lower intracellular antibiotic concentrations.

- In *K. pneumoniae*, overproduction of the AcrAB-TolC efflux pump and *ramR* mutations (a regulator of acrAB) have been described (Başaran and Öksüz, 2025; Satapoomin et al., 2022).
- In *P. aeruginosa*, mutations in regulators of the MexAB-OprM efflux pump system have been detected in resistant strains (González-Pinto et al., 2024; Sastre-Femenia et al., 2025).

*Enzymatic inactivation*.

- KPC overproduction: elevated blaKPC expression, most commonly due to increased plasmid copy number or promoter mutations, leads to KPC enzyme overproduction. This saturates the available vaborbactam, allowing uninhibited KPC enzymes to hydrolyse meropenem (Boattini et al., 2024; Bovo et al., 2023; Başaran and Öksüz, 2025; Yasmin et al., 2024; Bongiorno et al., 2022; Gaibani et al., 2022e; Gaibani et al., 2022c; Sun et al., 2017; Rogers et al., 2023a; Findlay et al., 2023; Satapoomin et al., 2022; Vaiana et al., 2025).
- Other carbapenemases: resistance can also be mediated by enzymes not effectively inhibited by vaborbactam, including OXA-48-like carbapenemases (OXA-48, OXA-181, OXA-232) and MBLs (NDM-1, NDM-5) (Bovo et al., 2023; Bongiorno et al., 2022; Rogers et al., 2023a; Cienfuegos-Gallet et al., 2024; Di Marcantonio et al., 2024; Sader et al., 2024).

## Nitroimidazole antibiotics

*Overview.* Nitroimidazoles represent a long-standing class of antimicrobial agents, first introduced in the late 1950s with metronidazole. Initially developed for the treatment of *Trichomonas vaginalis* infections, they were later found to have a remarkably broad spectrum of activity. Today, they remain widely used for the management of anaerobic bacterial infections, as well as protozoal and some mycobacterial diseases (see Table 4).

**Table 4:** Nitroimidazoles mechanisms of action and resistance.

Nitroimidazoles	
Mechanism of action	Resistance mechanisms
Prodrugs activated under anaerobic or microaerophilic conditions via microbial nitroreductases, generating cytotoxic radicals that cause DNA strand breaks and disrupt nucleic acid synthesis	Reduced activation due to decreased expression or activity of nitroreductases and other oxidoreductases
	Mutations in genes involved in electron transport and reductive pathways
	Enhanced oxidative stress responses, limiting formation of toxic intermediates

*Mechanism of action.* Nitroimidazoles are prodrugs that require intracellular activation under anaerobic or microaerophilic conditions. Their nitro group is reduced by microbial nitroreductases, generating short-lived, cytotoxic intermediates (such as nitroradical anions) that cause DNA strand breaks and damage to essential macromolecules, ultimately leading to cell death. Although the precise molecular events are not fully elucidated, disruption of nucleic acid synthesis is considered a key mechanism (Jenks, 2010).

*Resistance mechanism.* Since their activity relies on intracellular reduction of the nitro group, resistance generally arises from decreased expression or activity of the reductive enzymes involved in drug activation. Mutations in oxidoreductase genes, alterations in electron transport pathways, and enhanced oxidative stress responses have all been implicated in resistance. As a result, resistant strains exhibit impaired drug activation and reduced intracellular accumulation of toxic intermediates (Alauzet et al., 2019).

### Pretomanid

*Overview.* Pretomanid is a novel nitroimidazole derivative developed for the treatment of tuberculosis. It is a prodrug activated by the F_420_ cofactor system in *Mycobacterium tuberculosis*. Once activated, it exerts bactericidal activity both under aerobic conditions—by inhibiting mycolic acid biosynthesis—and under anaerobic conditions—by releasing reactive nitrogen species that damage essential cellular components.

*Clinical use.* Pretomanid is primarily indicated for the treatment of multidrug-resistant tuberculosis (MDR-TB) and rifampicin-resistant tuberculosis (RR-TB). According to WHO guidelines, it is administered in combination therapy, most notably as part of the BPaLM regimen (bedaquiline, pretomanid, linezolid, moxifloxacin), which has shown superior efficacy and safety compared with traditional MDR-TB regimens.

*Resistance mechanisms.* Resistance to pretomanid arises almost exclusively from genetic alterations that impair drug activation via the F_420_ pathway.

*Mutations in ddn*. The *ddn* gene encodes the nitroreductase directly responsible for pretomanid activation. Mutations such as Trp20Stop, W88Stop, L107P, G53D, and deletions prevent activation and confer resistance (Liu et al., 2022; Trisakul et al., 2022; Negi et al., 2024; Nguyen et al., 2023; Rifat et al., 2020; Gómez-González et al., 2021; Mansjö et al., 2022; Rossini and Dias, 2023).

*Mutations in fdg1*. Fgd1 maintains reduced F_420_ levels. Frameshift (G49fs) and binding site mutations disrupt this function, lowering pretomanid activity (Liu et al., 2022; Negi et al., 2024; Nguyen et al., 2023; Rifat et al., 2020; Gómez-González et al., 2021; Rossini and Dias, 2023; Purwantini et al., 2018).

*Mutations in fbiA, fbiB, fbiC, and fbiD*. These genes are involved in F_420_ biosynthesis. Codon stops, amino acid substitutions, and indels have been identified in resistant strains (Liu et al., 2022; Negi et al., 2024; Nguyen et al., 2023; Rifat et al., 2020; Gómez-González et al., 2021; Rossini and Dias, 2023; Purwantini et al., 2018).

*Putative additional mechanisms.* Mutations in rv0078 and rv2073c have been described, though their role in resistance remains uncertain (Yan et al., 2024). Loss of CinA increases susceptibility, suggesting its role as a resistance determinant (Kreutzfeldt et al., 2022). Cross-resistance between pretomanid and metronidazole has also been noted, possibly due to shared activation pathways (Conkle-Gutierrez et al., 2024).

## Pleuromutilins

*Overview.* Pleuromutilins are a class of antibiotics originally derived from basidiomycete fungi in the genus *Clitopilus*, notably *Clitopilus passeckerianus* (which was historically classified as *Pleurotus mutilus* or *P. passeckerianus*), in the 1950s. While early compounds were mainly developed for veterinary use, interest in pleuromutilins for human medicine has increased in recent decades. Retapamulin was the first pleuromutilin approved for topical use in humans, and lefamulin became the first pleuromutilin approved for systemic use in 2019 (see Table 5).

**Table 5:** Pleuromutilins mechanisms of action and resistance.

Pleuromutilins	
Mechanism of action	Resistance mechanisms
Bind the peptidyl transferase centre of the 50S ribosomal subunit, interfering with A and P sites and blocking peptide bond formation	Efflux pump overexpression
	Target-site mutations in rRNA or ribosomal proteins that alter the binding pocket
	Generally low resistance frequency due to the conserved, unique binding site

*Mechanism of action.* Pleuromutilins inhibit bacterial protein synthesis by binding to the peptidyl transferase centre of the 50S ribosomal subunit. This binding interferes with both the A and P sites of the ribosome, preventing peptide bond formation and halting protein synthesis. The highly specific binding explains their low propensity for cross-resistance with other classes of protein synthesis inhibitors.

*General resistance mechanism.* Resistance to pleuromutilins is considered uncommon due to the unique ribosomal binding pocket and the conserved nature of the target site. However, potential mechanisms include efflux pump overexpression and mutations in ribosomal proteins or rRNA that alter the drug-binding site. Because of their novel mechanism, pleuromutilins retain activity against many multidrug-resistant pathogens.

### Lefamulin

*Overview.* Lefamulin exhibits a broad spectrum of activity with potent efficacy against Gram-positive pathogens, key atypical pathogens, and some Gram-negative respiratory pathogens.

*Clinical use.* Lefamulin is approved for the treatment of CABP in adults. The main causative pathogens include *Streptococcus pneumoniae*, methicillin-susceptible *S. aureus* (MSSA), MRSA, *Haemophilus influenzae* (*H. influenzae*), *Legionella pneumophila*, *Chlamydophila pneumoniae*, and *Mycoplasma pneumoniae*.

*Resistance mechanisms.* Resistance to lefamulin can occur via two main mechanisms: efflux pump overexpression and mutations to the ribosomal target site.

*Increased efflux pump expression*. Efflux-mediated resistance has been observed in *S. aureus* and *H. influenzae*.

- In *S. aureus*, resistance is associated with the presence of efflux pump genes such as *vga(A)* and its variants, including *vga(E)* and *lsa(E).* These genes encode ABC transporters that can export pleuromutilins out of the bacterial cell (Paukner et al., 2024; Mendes et al., 2019).
- In *H. influenzae*, lefamulin-non-susceptible strains have shown premature stop codons in the *acrR* gene, which encodes the repressor of the AcrAB-TolC efflux system. This results in efflux pump overexpression and in a decreased intracellular concentration of lefamulin (Paukner et al., 2024).

*Mutations to the ribosomal target site*. Ribosomal mutations are the second main mechanism and have been identified in *Streptococcus* spp. and *H. influenzae*. These mutations involve alterations to the 23S rRNA and ribosomal proteins L3 (*rplC*) and L4 (*rplD*), disrupting drug binding and impairing inhibition of protein synthesis (Paukner et al., 2024; Mendes et al., 2019).

*Putative additional mechanisms.* Mutations in the L22 ribosomal protein have been detected in some *H. influenzae* strains, potentially contributing to resistance (Paukner et al., 2024).

## Tetracycline

*Overview.* Tetracyclines are a class of broad-spectrum antibiotics first introduced in the late 1940s. They possess activity against a wide range of pathogens, including Gram-positive and Gram-negative bacteria, spirochetes, rickettsiae, mycoplasmas, and some protozoa. This spectrum supports their use in various infections, such as rickettsioses, spirochaetal infections (e.g., Lyme disease), sexually transmitted infections (e.g., chlamydia), zoonoses (e.g., brucellosis), and respiratory tract infections (e.g., *M. pneumoniae*). They are also used for non-bacterial applications like malaria prophylaxis and are a mainstay in the long-term treatment of acne vulgaris due to their anti-inflammatory properties. However, their first-line clinical use for systemic infections has declined due to the global spread of resistance and significant adverse effects, including gastrointestinal toxicity, phototoxicity, and irreversible tooth discoloration in children (see Table 6).

**Table 6:** Tetracyclines mechanisms of action and resistance.

Tetracyclines	
Mechanism of action	Resistance mechanisms
Bind the 30S ribosomal subunit and block aminoacyl-tRNA entry, inhibiting protein elongation	Ribosomal protection proteins (Tet(M), Tet(O))
	Efflux pumps (Tet(A), Tet(K))
	Enzymatic inactivation (Tet(X))
	Widespread via plasmids/transposons

*Mechanism of action.* Tetracyclines interfere with bacterial protein synthesis by binding primarily to the 30S ribosomal subunit (with some evidence for additional interaction with the 50S subunit). They bind to the A-site, which prevents the aminoacyl-tRNA from attaching to the acceptor site on the ribosome. This effectively blocks the incorporation of new amino acids into the growing peptide chain, halting elongation.

*General resistance mechanism.* Resistance to tetracyclines is widespread and is primarily mediated by acquired genes located on mobile genetic elements like plasmids and transposons. These genes encode three main mechanisms:

- *Ribosomal protection proteins.* These proteins (e.g., Tet(M), Tet(O)) prevent tetracyclines from blocking protein synthesis by altering ribosome conformation.
- *Efflux pumps.* Active expulsion of tetracyclines from the bacterial cell via transporter proteins (e.g., Tet(A), Tet(K)) reduces intracellular concentrations below inhibitory levels.
- *Enzymatic inactivation.* Specific enzymes (e.g., Tet(X)) can chemically modify and inactivate tetracyclines.

### Omadacycline

*Overview.* Omadacycline is a modern aminomethylcycline, structurally derived from tetracyclines, with broad-spectrum activity against both Gram-positive and Gram-negative bacteria. It was developed to overcome common tetracycline resistance mechanisms, such as efflux and ribosomal protection.

*Clinical use.* Omadacycline is approved for the treatment of CABP and ABSSSI. It is active against many bacterial species, including drug-resistant pathogens.

*Resistance mechanisms.* Resistance to omadacycline can be attributed to four main mechanisms.

*Efflux pump overexpression*. In *A. baumannii*, the AdeABC efflux pump, regulated by the AdeRS two-component system, reduces intracellular accumulation of omadacycline and promotes multidrug resistance (Lee et al., 2020).

*Modification of target sites*. Mutations in the *rpsJ* gene, encoding ribosomal protein S10 of the 30S subunit, have been observed in *S. aureus*. These mutations reduce drug affinity for the ribosome and impair antibacterial activity (Chen et al., 2025; Huang et al., 2025).

*Ribosomal protection proteins*. The *tet(M)* gene in *S. aureus* encodes a ribosomal protection protein that blocks tetracycline binding, thereby allowing translation to proceed in the presence of the antibiotic (Chen et al., 2025; Wang et al., 2020).

*Enzymatic inactivation*. Several Gram-negative pathogens produce enzymes that degrade tetracyclines, including omadacycline. For instance, *K. pneumoniae* can carry plasmid-borne *tet(X4),* while *A. baumannii* harbours *tet(X3)*, and *P. aeruginosa* clinical isolates have been reported to encode *tet(X7)*. These flavin-dependent monooxygenases inactivate omadacycline and other modern tetracyclines (Gasparrini et al., 2020; Liu et al., 2024; Talsma et al., 2024).

### Eravacycline

*Overview.* Eravacycline is a fully synthetic fluorocycline antibiotic belonging to the tetracycline class. It demonstrates potent activity against a wide variety of Gram-negative and Gram-positive pathogens, including many multidrug-resistant organisms. Structural modifications were introduced to evade common tetracycline resistance mechanisms, but resistance has still emerged in several species through diverse molecular strategies.

*Clinical use.* Eravacycline is approved for the treatment of cIAI. Its broad activity makes it particularly valuable against multidrug-resistant Gram-negative pathogens, including *A. baumannii* and CRE, as well as Gram-positive organisms such as *S. aureus* and *Enterococcus* spp.

*Resistance mechanisms.* Resistance to eravacycline arises primarily through efflux, ribosomal modifications, and enzymatic inactivation.

*Efflux pump overexpression*. This is the most prevalent mechanism of eravacycline resistance, mediated by diverse pumps across species:

- In *K. pneumoniae*, the AcrAB-TolC system (regulated by *ramA*), along with OqxAB and MacAB pumps, reduces intracellular drug concentrations (García et al., 2024; Zheng et al., 2018; Zhao et al., 2025).
- In *A. baumannii*, mutations in *adeS* lead to constitutive overexpression of the AdeABC pump, expelling eravacycline and other antibiotics (Başaran and Öksüz, 2025; García et al., 2024; Chen et al., 2024; Li et al., 2024; Gautam et al., 2024; Shi et al., 2020).
- In enterococci (*E. faecium*, *E. faecalis*), resistance is linked to the Tet(L) efflux pump and the putative transporter RS00630 (Boukthir et al., 2020; Wen et al., 2020).
- In *S. aureus*, the MepB pump contributes to efflux, often in conjunction with Tet(K) and Tet(L) (Zeng et al., 2022; Wang et al., 2021).

*Modification of target sites*. Mutations in the *rpsJ* gene, encoding ribosomal protein S10 of the 30S subunit, alter the tetracycline binding site and reduce eravacycline affinity. Such mutations have been identified in *E. faecium*, *E. faecalis*, and *S. aureus* (Başaran and Öksüz, 2025; Boukthir et al., 2020; Wen et al., 2020). Mutations in 16S rRNA also impair binding, contributing to resistance (Wen et al., 2020). Although resistance rates in *S. pneumoniae* remain low (1.9% in a Chinese study), mutations in *rpsJ* and possibly *rpsC* have been implicated (Lei et al., 2025).

*Enzymatic inactivation*. *Several* Gram-negative pathogens carry *tet(X)* variants encoding flavin-dependent monooxygenases that oxidize and inactivate eravacycline. In *K. pneumoniae*, plasmid-borne *tet(X4)* has been detected (Liu et al., 2024). In *A. baumannii*, plasmid-encoded *tet(X3)* and *tet(X5)* contribute to resistance (Talsma et al., 2024; Wang et al., 2019).

*Putative additional mechanisms.* Of *significant* concern, some *A. baumannii* strains harbour both *tet(X)* variants and carbapenemase genes (*bla*_NDM-1_, *bla*_OXA-97_, *bla*_OXA-23_), suggesting a potential link between tetracycline and carbapenem resistance (Talsma et al., 2024; Li et al., 2024).

## Aminoglycosides

*Overview.* Aminoglycosides are a class of antibiotics discovered in the 1940s, with streptomycin being the first to be used clinically. They are bactericidal agents with activity against a broad range of Gram-negative and certain Gram-positive bacteria. They exhibit concentration-dependent killing and a significant post-antibiotic effect, making them suitable for once-daily dosing regimens. Aminoglycosides remain essential in the treatment of severe infections, such as septicemia, pneumonia, endocarditis, cUTIs, cIAIs, and tuberculosis. Their clinical use is often limited by nephrotoxicity and ototoxicity, which require careful therapeutic monitoring (see Table 7).

**Table 7:** Aminoglycosides mechanisms of action and resistance.

Aminoglycosides	
Mechanism of action	Resistance mechanisms
Bind 16S rRNA of 30S ribosomal subunit, causing misreading of mRNA and production of non-functional proteins	Enzymatic inactivation by aminoglycoside-modifying enzymes (AAC, ANT, APH)
Induce conformational changes at the ribosomal A-site, disrupting protein synthesis and bacterial membrane integrity	Target modification: mutations in 16S rRNA or ribosomal proteins
	Reduced intracellular accumulation via decreased uptake or efflux pump overexpression

*Mechanism of action.* Aminoglycosides act through two primary mechanisms:

- Binding to the 16S rRNA of the 30S ribosomal subunit, interfering with accurate decoding of the mRNA template.
- Inducing conformational changes at the ribosomal A-site, leading to misincorporation of amino acids and the production of non-functional proteins. The incorporation of these misfolded proteins into the bacterial cell membrane disrupts its integrity, contributing to the drug’s rapid bactericidal effect. Their selectivity arises from structural differences between bacterial and eukaryotic ribosomes.

*General resistance mechanism.* Resistance to aminoglycosides develops primarily through three strategies:

- *Drug inactivation.* The most common mechanism involves aminoglycoside-modifying enzymes (AMEs), such as acetyltransferases (AAC), nucleotidyltransferases (ANT), and phosphotransferases (APH). These enzymes chemically modify the antibiotic, preventing its interaction with the ribosome.
- *Target modification.* Mutations in the 16S rRNA or ribosomal proteins reduce drug affinity for the ribosome, impairing aminoglycoside binding.
- *Reduced intracellular accumulation.* This can occur through mutations that decrease the energy-dependent uptake of the drug or through the action of efflux pumps that actively export aminoglycosides out of the cell.

### Plazomicin

*Overview.* As a next-generation aminoglycoside, plazomicin was specifically designed to overcome the common resistance mechanisms that limit traditional aminoglycosides, particularly aminoglycoside-modifying enzymes (AMEs). It retains activity against MDR *Enterobacterales* and some non-fermenters, including carbapenemase-producing strains. Its chemical modifications confer stability against many aminoglycoside-modifying enzymes, making it a valuable option where older aminoglycosides fail.

*Clinical use.* Plazomicin is approved for the treatment of cUTI and has been evaluated for bloodstream infections and VAP. It exhibits potent *in vitro* activity against multidrug-resistant *Enterobacterales*, including *E. coli* and *K. pneumoniae*, and has variable activity against non-fermenters like *A. baumannii* and *P. aeruginosa*. It also retains activity against *S. aureus*. Due to its critical role in treating severe infections caused by resistant bacteria, plazomicin is included in the WHO list of essential medicines and categorized as a “reserve” antibiotic, to be used only in severe or last-resort cases.

*Resistance mechanisms.* Despite its structural modifications, resistance to plazomicin has emerged through three main mechanisms.

*Modification of target sites*. The most relevant mechanism is mediated by 16S rRNA methylases, which add a methyl group to specific residues (commonly G1405) of the 16S rRNA in the 30S ribosomal subunit. This prevents plazomicin and other aminoglycosides from binding to their target. Genes encoding these enzymes (e.g., *rmtC*, *rmtF1*, *rmtB1*, *armA*, *rmtF*, *rmtB*) have been widely reported in *E. coli* and *K. pneumoniae* (Dahdouh et al., 2024; Teo et al., 2023; Arca-Suárez et al., 2022; Wachino et al., 2020; Gür et al., 2020; Jacobs et al., 2020; Galani et al., 2019; Walkty et al., 2019; Castanheira et al., 2018; Doi et al., 2016).

*Enzymatic inactivation.* The aminoglycoside 2′-acetyltransferase encoded by *aac*(2′)-*Ia* acetylates plazomicin, reducing its affinity for the ribosome. This mechanism is often found alongside other AMEs (e.g., AAC(3′)-IIa, APH(3′)-Ia) in resistant isolates, contributing to a broader profile of cross-resistance to other aminoglycosides (Ngo et al., 2023; Bassenden et al., 2021; Almaghrabi et al., 2014).

*Co-occurrence with carbapenemases.* Plazomicin resistance is frequently linked with carbapenemase production in *Enterobacterales*. Enzymes such as NDM, VIM-1, OXA-1, OXA-181, OXA-48, and OXA-232 have been reported in clinical isolates, resulting in multidrug- or even pan-drug-resistant strains (Dahdouh et al., 2024; Arca-Suárez et al., 2022; Gür et al., 2020; Jacobs et al., 2020; Galani et al., 2019; Castanheira et al., 2018; Öztaş et al., 2024; Fleischmann et al., 2020).

*Putative additional mechanisms.* A recently described 16S rRNA methyltransferase, NpmC, modifies residue A1408 and confers resistance even to novel aminoglycosides such as plazomicin. Although currently found mainly in non-pathogenic bacteria, its potential for horizontal transfer poses a clinical threat (Matamoros et al., 2025).

## Rifamycin

*Overview.* Rifamycins are ansamycin antibiotics (e.g., rifampicin/rifampin, rifabutin, rifapentine, rifaximin). They are bactericidal cornerstone agents in tuberculosis regimens (used in combination) and are also active against various Gram-positive and Gram-negative bacteria; the non-absorbed rifaximin is used for intestinal infections such as traveller’s diarrhoea (see Table 8).

**Table 8:** Rifamycins mechanisms of action and resistance.

Rifamycins	
Mechanism of action	Resistance mechanisms
Bind the β subunit of DNA-dependent RNA polymerase (RpoB), inhibiting transcription initiation and RNA synthesis	Target modification: mutations in rpoB, especially in the RRDR
	Reduced intracellular accumulation via efflux pumps (less common)
	Enzymatic inactivation (e.g., ADP-ribosylation by Arr enzymes; less common)

*Mechanism of action.* They inhibit bacterial RNA synthesis by binding with high affinity to the *β* subunit of DNA-dependent RNA polymerase (RpoB), blocking transcription initiation and leading to rapid bacterial killing (particularly against mycobacteria). Their selectivity arises from the minimal effect on eukaryotic RNA polymerase.

*General resistance mechanism.* The principal mechanism is target alteration via mutations in the *rpoB* gene (notably within the rifampicin-resistance–determining region (RRDR)), which reduces drug binding. The high spontaneous mutation rate in this gene is the reason rifamycins must always be used in combination therapy. Less commonly, efflux and enzymatic inactivation (e.g., ADP-ribosylation by Arr enzymes in some species) contribute to decreased susceptibility.

### Rifamycin SV

*Overview.* Rifamycin SV (commercial name Aemcolo) is a specific molecule within the rifamycin class. It is distinct from the more widely known rifampicin (also known as rifampin), which is a semi-synthetic derivative. While they share the class’s core mechanism of action—inhibition of bacterial RNA polymerase—their clinical applications differ significantly. Rifamycin SV is formulated with a delayed-release system that ensures local delivery in the colon, making it suited for gastrointestinal infections. It is approved for the treatment of traveler’s diarrhea caused by non-invasive strains of *E. coli*.

*Clinical use.* Rifamycin SV is indicated for the treatment of traveler’s diarrhea. Clinical studies have demonstrated it to be as effective as ciprofloxacin, with the added benefit of a lower risk of selecting for ESBL-producing *E. coli* (Kantele and Lääveri, 2022). Its use is restricted to non-systemic infections due to its minimal systemic absorption, which avoids widespread exposure and reduces the risk of collateral damage (e.g., disruption of gut microbiota and selection of resistance in systemic pathogens).

*Resistance mechanisms.* Resistance to rifamycin SV in *E. coli* has been investigated mainly in strains isolated from farms, though some human isolates have also shown resistance (Castillo et al., 2022). The mechanisms mirror those of the broader class but have been characterized for this specific drug.

*Enzymatic inactivation.* The *arr-3* gene, identified in *E. coli* isolates from dairy farms, encodes an ADP-ribosyltransferase capable of inactivating rifamycins (Al-Mustapha et al., 2023; Shoaib et al., 2024).

*Efflux pumps.* Resistance has also been associated with overexpression of multidrug efflux systems. Genes such as *marA*, *acrA*, *acrB*, *tolC*, and *acrE*—key components of the AcrAB-TolC efflux system—were upregulated in resistant *E. coli* from cattle (Al-Mustapha et al., 2023). These systems reduce intracellular concentrations of rifamycin SV, contributing to resistance and cross-resistance with other antimicrobials.

## Quinolones and fluoroquinolones

*Overview.* Quinolones are a class of synthetic antibacterial agents. The initial compounds were developed to target Gram-negative bacteria, but the addition of a fluorine atom led to the development of fluoroquinolones, which exhibit a broader spectrum, improved pharmacokinetics, and expanded clinical utility. Their clinical use is often categorized by generation, which reflects their evolving spectrum (see Table 9):

- *First-generation:* used for uncomplicated UTIs (mostly withdrawn).
- *Second-generation:* enhanced Gram-negative (including *P. aeruginosa*) activity.
- *Third-generation:* improved activity against *S. pneumoniae*.
- *Fourth-generation:* broad-spectrum coverage, including anaerobes.
- *Fifth-generation:* developed to target multidrug-resistant pathogens like MRSA and resistant *S. pneumoniae*.

**Table 9:** Quinolones and fluoroquinolones mechanisms of action and resistance.

Quinolones and fluoroquinolones	
Mechanism of action	Resistance mechanisms
Inhibit bacterial type II topoisomerases (DNA gyrase and topoisomerase IV), causing double-strand DNA breaks and replication arrest	Target site mutations in QRDRs of *gyrA*, *gyrB*, *parC*, *parE*
	Reduced intracellular accumulation via efflux pump overexpression or porin loss
	Plasmid-mediated resistance (e.g., *qnr*, *aac(6′)-Ib-cr*)

Despite their broad use, safety concerns (e.g., tendonitis, neuropathy) and widespread resistance have led to restrictions on their use.

*Mechanism of action.* Quinolones and fluoroquinolones exert bactericidal effects by inhibiting bacterial type II topoisomerases: (i) DNA gyrase, which introduces negative supercoils into DNA, essential for replication and transcription, and (ii) topoisomerase IV, which separates interlinked daughter chromosomes after replication. Inhibition of these enzymes causes double-strand DNA breaks, leading to replication arrest and cell death. The primary target varies by species; DNA gyrase is typically primary in Gram-negative bacteria, while topoisomerase IV is often primary in Gram-positive organisms.

*General resistance mechanism.* Resistance arises through several mechanisms, which often occur synergistically.

*Target site modifications.* Mutations in the Quinolone Resistance-Determining Regions (QRDRs) of the genes encoding DNA gyrase (*gyrA*, *gyrB*) and topoisomerase IV (*parC*, *parE*) are the most common chromosomal mechanisms. These mutations reduce drug binding affinity.

*Reduced intracellular accumulation.* This can occur through overexpression of efflux pumps (e.g., in *P. aeruginosa*) or loss of porins in the outer membrane, both of which decrease the intracellular drug concentration.

*Plasmid-mediated resistance*. This increasingly prevalent mechanism involves genes such as *qnr* (which encodes a protein that protects the target enzymes) and *aac(6′)-Ib-cr* (which encodes an enzyme that modifies and inactivates certain fluoroquinolones).

### Delafloxacin

*Overview.* Delafloxacin is a novel fluoroquinolone antibiotic with a distinct chemical structure that confers potent activity against a broad spectrum of Gram-positive and Gram-negative bacteria. Its unique properties enhance its stability and efficacy in acidic environments (e.g., abscesses) and favour intracellular accumulation. Like other fluoroquinolones, it is a bactericidal agent that inhibits bacterial type II topoisomerases (DNA gyrase and topoisomerase IV) interfering with DNA replication and transcription. Its balanced activity against both enzymes complicates the emergence of resistance.

*Clinical use.* Delafloxacin is approved for the treatment of ABSSSI and CABP in adults. Its broad spectrum includes key pathogens such as MRSA, *E. coli*, *K. pneumoniae*, and *S. pneumoniae*. Its enhanced activity in acidic tissues provides a potential therapeutic advantage for infections like abscesses where the pH is low.

*Resistance mechanisms.* Although resistance to delafloxacin remains relatively uncommon due to its dual targeting, it can emerge through mechanisms shared across the fluoroquinolone class, often acting in combination.

*Increased efflux pump expression*. Overexpression of efflux systems is a significant resistance mechanism, reducing intracellular drug concentrations.

- In *K. pneumoniae*, the overexpression of the OqxAB and AcrAB contributes to resistance (Kubicskó et al., 2025).
- In *P. aeruginosa*, mutations in the *mexR* lead to overexpression of the MexAB-OprM (Robert et al., 2025).
- In *E. coli*, mutations in regulatory genes activate the ACRAB-TolC and MDRABC-TolC (Kubicskó et al., 2024; Gulyás et al., 2023).
- In *S. aureus,* plasmid encoding pumps like QacC enhance resistance (Bolaños et al., 2024; de la Rosa et al., 2023; Byrd et al., 2021).
- Efflux mechanisms often act synergistically with target site mutations, substantially increasing the risk of therapeutic failure.

*Target site modification.* Mutations in the quinolone resistance-determining regions (QRDRs) of the genes encoding DNA gyrase (*gyrA*, *gyrB*) and topoisomerase IV (*parC*, *parE*) are the primary mechanism. These mutations reduce drug-binding affinity and have been documented in key pathogens:

- *K. pneumoniae*: Mutations in *gyrA* and *parC* (Kubicskó et al., 2025).
- *P. aeruginosa*: Mutations in *gyrA*, *gyrB*, *parC*, and *parE* (Robert et al., 2025).
- *E. coli*: Initial mutations in *gyrA* (e.g., at codons Ser83, Asp87) confer low-level resistance (Kubicskó et al., 2024; Gulyás et al., 2023).
- *S. aureus*: Mutations in *gyrA* and *parC* confer cross-resistance within the class (Kubicskó et al., 2025).

## Triazaacenaphthylenes

*Overview*. Triazaacenaphthylenes represent a novel class of antibacterial agents characterized by their unique tricyclic core structure. This chemical class is distinct from all other approved antibiotics, including the quinolones. Gepotidacin is the first approved member of this class. These agents were designed to inhibit bacterial type II topoisomerases through a mechanism distinct from fluoroquinolones, aiming to overcome fluoroquinolone resistance and avoid off-target human effects (see Table 10).

**Table 10:** Triazaacenaphthylenes mechanisms of action and resistance.

Triazaacenaphthylenes	
Mechanism of action	Resistance mechanisms
Inhibit bacterial type II topoisomerases (DNA gyrase and topoisomerase IV) by binding a site distinct from fluoroquinolones, stabilizing single-stranded DNA breaks and blocking DNA replication	Target site mutations in *gyrA* and *parC* (overlapping or adjacent to QRDRs)
	Efflux pump overexpression (e.g., MtrCDE in *N. gonorrhoeae*, AcrAB-TolC in *Enterobacterales*)
	Combined mechanisms: low-level efflux plus target site mutations can produce clinically relevant resistance

*Mechanism of action*. Triazaacenaphthylenes are bactericidal. They exert their effect by inhibiting Type II topoisomerases, including bacterial topoisomerase II (DNA gyrase) and topoisomerase IV, thereby inhibiting DNA replication. Crucially, they bind to a different site on the enzyme-DNA complex than fluoroquinolones, stabilizing a single-stranded DNA break. This novel mechanism results in a different resistance profile and maintained activity against many fluoroquinolone-resistant strains.

*General resistance mechanism*. As a new class, surveillance is ongoing. Based on its target, anticipated and observed mechanisms include:

- *Target site modifications*: Mutations in the genes encoding the target enzymes, particularly in the *gyrA* and *parC* subunits, can reduce drug binding and confer resistance. These mutations may occur in regions overlapping with or adjacent to the quinolone resistance-determining regions (QRDRs).
- *Efflux pump overexpression*: Activation of endogenous bacterial efflux pumps (e.g., MtrCDE in *N. gonorrhoeae*, AcrAB-TolC in *Enterobacterales*) can reduce intracellular drug accumulation.
- *Combined mechanisms*: The confluence of multiple low-level resistance mechanisms (e.g., modest efflux plus a target site mutation) can lead to clinically significant resistance.

### Gepotidacin

*Overview*. Gepotidacin is a novel triazaacenaphthylene antibiotic and the first in its class to be approved for clinical use. It was developed specifically to be effective against pathogens resistant to existing antibiotics, including fluoroquinolones, and to treat infections caused by both Gram-positive and Gram-negative bacteria (Keam, 2025).

*Clinical use*. Gepotidacin is approved for the treatment of uncomplicated urinary tract infections (uUTI) caused by *E. coli* and *S. saprophyticus*, and for uncomplicated urogenital gonorrhea caused by *N. gonorrhoeae*. Its oral formulation and novel mechanism make it a valuable new option for these common outpatient infections.

*Resistance mechanisms*. Although gepotidacin is a novel antibiotic, emerging resistance mechanisms have already been reported, particularly in *N. gonorrhoeae* and *Enterobacterales*.

- *Target site modifications*. The most critical mechanism involves mutations in the *gyrA* and *parC* genes, encoding subunits of DNA gyrase and topoisomerase IV, respectively. While single substitutions confer only modest changes in susceptibility, dual mutations (e.g., GyrA D82N combined with ParC D79N) lead to high-level resistance and were linked to reduced clinical efficacy in trial settings (Szili et al., 2019).
- *Efflux pump overexpression*. Up-regulation of efflux (e.g., MtrCDE in *N. gonorrhoeae*; AcrAB-TolC in *Enterobacterales*) lowers intracellular drug levels and augments target-site mutations.
- *Putative additional mechanisms.* Although gepotidacin hits the same enzymes as fluoroquinolones, its different binding site means many fluoroquinolones-resistant strains remain susceptible (Biedenbach et al., 2016); however, prior fluoroquinolone exposure may co-select mutations affecting both classes, warranting stewardship (Szili et al., 2019).

## Key insights and implications for public health and antimicrobial stewardship

The escalating threat of antimicrobial resistance (AMR) remains a paramount challenge to global public health. Although the period from 2017 to 2025 saw significant advances in antibiotic development, the rapid and simultaneous emergence of sophisticated resistance mechanisms against these new drugs underscores the remarkable adaptability of bacterial pathogens. In this review, we summarize how WHO-listed priority pathogens evade recently developed antibiotics, focusing on target site mutations (e.g., PBP3, RpoB, QRDRs), efflux pump overexpression (e.g., AdeABC, AcrAB-TolC), and enzymatic inactivation (e.g., Tet(X) variants, 16S rRNA methyltransferases).

The evidence demonstrates that resistance evolves quickly, often circumventing even the most advanced therapeutic options through these genetic and biochemical strategies. Crucially, resistance is often more than a simple, one-gene event. As detailed for agents like cefiderocol and the novel β-lactam combinations, resistance often arises from a complex interplay of multiple chromosomal mutations affecting permeability, efflux, and target sites, sometimes combined with acquired enzymes. This polygenic basis complicates resistance prediction and diagnosis, exposing the limits of current detection systems. Importantly, resistance determinants are also emerging in species not routinely exposed to some of these antibiotics. For instance, plasmid-mediated β-lactamases reducing susceptibility to β-lactam/β-lactamase inhibitor combinations have been detected in *Serratia marcescens* and *Morganella morganii* (Sabtcheva et al., 2024). Likewise, a recent survey of wastewater from six German tertiary-care hospitals identified 97 cefiderocol-resistant isolates across species such as *Enterobacter roggenkampii*, *Klebsiella oxytoca*, *S. marcescens*, and *Citrobacter farmer* (Erler et al., 2025). These isolates frequently carried multiple β-lactamase and carbapenemase genes, heavy-metal and biocide resistance determinants, and diverse plasmids, highlighting their role as potential multidrug-resistance reservoirs outside the WHO priority pathogen list.

These observations underscore the urgent need for a sustained and coordinated global effort to mitigate the impact of AMR. Addressing this challenge requires a true ‘One Health’ approach, combining new drug development with strengthened surveillance, stewardship, and infection control. In this context, the complexity of the resistance landscape detailed herein reveals the limitations of our current diagnostic paradigms. If we are to preserve the efficacy of both existing and newly developed antimicrobials, our ability to detect and predict resistance must evolve to match the speed and sophistication of bacterial evolution. This imperative leads to the critical role of next-generation technologies in redefining AMR diagnosis and surveillance.

## Diagnosis of AMR: the role of bioinformatics and artificial intelligence

Rapid and accurate detection of AMR is central to effective therapy, infection control, and antimicrobial stewardship. While phenotypic antimicrobial susceptibility testing (AST) remains the clinical standard, whole-genome sequencing (WGS) has emerged as a powerful complementary approach, especially thanks to the long-read sequencing technologies (e.g., PacBio, Oxford Nanopore) that enable the complete assembly of bacterial genomes and plasmids (Lavezzo et al., 2016): when paired with robust computational analysis, WGS holds the promise of providing faster, more comprehensive resistance predictions across both known and novel agents.

Traditional genotype-based methods for AMR prediction rely on curated databases of known resistance determinants, such as horizontally acquired resistance genes or well-characterized chromosomal mutations (Gupta et al., 2014; Alcock et al., 2020; Florensa et al., 2022). These tools are effective when resistance is conferred by known, well-documented mechanisms. However, many resistance phenotypes, especially against recently approved antibiotics, are polygenic, involve combinations of chromosomal mutations, regulatory changes, or non-canonical mechanisms not yet included in reference databases. In such cases, genotype-only approaches may underpredict resistance risk.

Machine learning (ML) and artificial intelligence (AI) are increasingly explored as complementary tools to overcome these limitations. ML models trained on paired genomic and phenotypic (AST) data can, in principle, learn complex, non-linear associations across the genome, including epistatic interactions, regulatory mutations, or combined effects of multiple loci, that escape standard presence/absence screening.

For instance, Ren et al. (2022) demonstrated that ML-based models could predict resistance phenotypes with improved accuracy compared to database-only approaches, by leveraging features beyond well-known determinants. Similarly, deep-learning and transfer-learning strategies (e.g., MSDeepAMR) have recently been proposed to enhance generalizability across different antibiotic classes, showing promising performance in exploratory studies (López-Cortés et al., 2024). In the context of *Mycobacterium tuberculosis*, Babirye et al. (2024) reported cross-dataset validation of ML predictions of drug susceptibility, underscoring the potential for ML to be applied across diverse bacterial species. Recent methodological advances further refine ML-based AMR prediction by applying explainability frameworks and careful benchmarking to improve interpretability and reduce overfitting (Valavarasu et al., 2025). Nonetheless, despite these advances, ML-based AMR diagnostics remain largely at the proof-of-concept stage rather than in widespread clinical use. Key challenges include:

- *Data quality, bias, and phenotypic heterogeneity*: models are only as good as the data they are trained on; limited geographical or lineage diversity in training datasets can compromise generalizability, while differences in AST platforms, breakpoint standards, and laboratory procedures introduce phenotypic variability that adds noise and hampers reproducibility across studies.
- *Lack of prospective clinical validation*: few studies have evaluated ML predictions in real-world clinical microbiology workflows, and data on sensitivity, specificity, and error rates remain scarce.
- *Interpretability*: complex models, especially deep-learning ones, often behave as “black boxes,” complicating interpretation and clinical trust.
- *Feature representation and standardization*: ML models rely on heterogeneous genomic feature sets (e.g., k-mers, SNPs, gene presence/absence), which complicates benchmarking, limits cross-dataset transferability, and hampers comparison between methods.
- *Infrastructure requirements*: high-throughput sequencing, bioinformatics expertise, computational resources, and standardized pipelines are not yet universally available across laboratories.

In light of current evidence, ML and AI should be viewed as promising complementary tools for AMR diagnosis, not as replacements for phenotypic testing or curated databases. Their greatest value lies in the potential identification of novel candidate resistance mechanisms that merit experimental validation, in augmenting databases with data-driven insights, and in enabling high-throughput, genome-scale prediction of polygenic or non-canonical resistance across large and diverse bacterial datasets, especially for newer antibiotics still lacking extensive phenotypic legacy data.

To achieve this goal, several technical and organizational obstacles must be addressed with internationally shared guidelines. A critical prerequisite for these AI-driven approaches to become robust and clinically actionable is the adoption of shared standards for data collection, representation, and model evaluation. In particular, global efforts should embrace the FAIR data principles (data being Findable, Accessible, Interoperable, and Reusable),^2^ to ensure that genomic and phenotypic datasets generated across laboratories and surveillance programmes can be systematically integrated, compared, and reused. This requires not only open, persistent identifiers and machine-readable metadata describing isolate characteristics, sequencing platforms, AST methodology, and resistance phenotypes, but also harmonized ontologies and controlled vocabularies to minimize ambiguity and batch effects. Equally important, the design, training, and evaluation of AI methods should follow transparent and reproducible standards (Matschinske et al., 2021) that include rigorous preprocessing, well-defined splitting strategies preventing data leakage, external cross-dataset validation, and systematic reporting of performance metrics and error rates. Establishing such FAIR-compliant data infrastructures and methodological guidelines will be essential to mitigate bias, support global benchmarking, and accelerate translation of AI-enabled AMR prediction from exploratory research into reliable clinical and public-health tools.

Realizing this potential will ultimately depend on coordinated global efforts to assemble large, diverse genomic–phenotypic resources and to validate AI models through rigorous external benchmarking and prospective clinical studies.

## Footnotes

1
https://www.ema.europa.eu/en/search

2
https://www.go-fair.org/fair-principles/

## Glossary

ABSSSI: Acute Bacterial Skin and Skin Structure Infections
AMR: Antimicrobial Resistance
AST: Antimicrobial Susceptibility Testing
BLIC: β-Lactam/β-Lactamase Inhibitor Combination
BPaLM: Bedaquiline, Pretomanid, Linezolid, Moxifloxacin
CABP: Community-Acquired Bacterial Pneumonia
cIAI: Complicated Intra-Abdominal Infection
CRE: Carbapenem-Resistant Enterobacterales
cUTI: Complicated Urinary Tract Infection
CABP: Community-Acquired Bacterial Pneumonia
EMA: European Medicines Agency
ESBL: Extended-Spectrum Beta-Lactamase
FAIR: Findable, Accessible, Interoperable, and Reusable
FDA: Food and Drug Administration (U.S.)
HABP: Hospital-Acquired Bacterial Pneumonia
MDR: Multidrug-Resistant
MDR-TB: Multidrug-Resistant Tuberculosis
MRSA: Methicillin-Resistant *Staphylococcus aureus*
MSSA: Methicillin-Susceptible *Staphylococcus aureus*
PBP: Penicillin-Binding Protein
QRDR: Quinolone Resistance-Determining Region
RR-TB: Rifampicin-Resistant Tuberculosis
SAB: *Staphylococcus aureus* Bacteremia
VABP: Ventilator-Associated Bacterial Pneumonia
WHO: World Health Organization
AME: Aminoglycoside-Modifying Enzyme
MBL: Metallo-β-Lactamase
KPC: *Klebsiella pneumoniae* Carbapenemase
NDM: New Delhi Metallo-β-lactamase
VIM: Verona Integron-encoded Metallo-β-lactamase
IMP: Imipenemase Metallo-β-lactamase
OXA: Oxacillinase
TB: Tuberculosis
uUTI: Uncomplicated Urinary Tract Infection
