# Correction: Molecular resistance mechanisms to newly approved antibiotics (2017–2025) in WHO priority pathogens

**DOI:** 10.3389/fmicb.2026.1793977

**Published:** 2026-02-13

**Authors:** Frontiers Production Office

**Affiliations:** Frontiers Media SA, Lausanne, Switzerland

**Keywords:** antimicrobial resistance (AMR), molecular diagnostics, novel antibiotics, resistance mechanisms, WHO priority pathogens

The hierarchical structure of sections and sub-sections were incorrectly formatted in the final version of the article.

- The bullet points starting at “In *E. coli*, expression of the AmpC…” are intended as sub-sections of **“*Enzymatic inactivation*”**, page 4, Aztreonam-avibactam.
- The sections starting at “*Alterations in iron transport systems.”* are intended as sub-sections of **“Resistance mechanisms”**, page 5, Cefiderocol.
- The sections starting at “*A. baumannii*:” are sub-subsections of the section “***Alterations in iron transport systems*”**, page 6, Cefiderocol.
- Similarly, the sections starting at “*Reduced outer membrane permeability*.” are sub-sections of **“Resistance mechanisms”**, page 7, Meropenem-vaborbactam.
- The sections at “OmpK35 inactivation:” are sub-sections of the “***K*.** ***pneumoniae*”**, page 7, Meropenem-vaborbactam.
- Sections beginning at lines “*Mutations in ddn”* are sub-sections of **“Resistance mechanisms”**, page 8, Pretomanid.
- Sections beginning at “*Increased efflux pump expression”* are sub-sections of **“Resistance mechanisms”**, page 8, Lefamulin.
- Sections beginning at lines “*Efflux pump overexpression*” are sub-sections of **“Resistance mechanisms”**, page 9, Omadacycline.
- Sections beginning at lines “*Efflux pump overexpression*” are sub-sections of **“Resistance mechanisms”**, page 9, Eravacycline.
- Sections beginning at “*Modification of target sites*” are sub-sections of **“Resistance mechanisms”**, page 10, Plazomicin.
- Sections beginning at “*Enzymatic inactivation*” are sub-sections of **“Resistance mechanisms”**, page 11, Rifamycin SV.
- Sections beginning at “*Target site modifications*” are sub-sections of **“General resistance mechanisms”**, page 12, Quinolones and fluoroquinolones.
- Sections beginning at “*Increased efflux pump expression*” are sub-sections of **“Resistance mechanisms”**, page 12, Delafloxacin.

There was a mistake in Table 2 as published. In line 2 of the table, regarding “Cefepime and enmetazobactam” antibiotic, the column “Main use” reports “HABP, cUTIs, and cIAIs”. The corrected Table 2 appears below, where “cIAIs” has been removed for that drug.

**Table 2:** Antimicrobial drugs approved by FDA and/or EMA since 2017.

**Entry**	**Drug name**	**Approval year**	**Drug class (*subclass*)**	**Main use**
1.	Aztreonam and avibactam	2024 EMA	β-lactams and BLICs *Monobactam and non-β-lactam β-lactamase inhibitor*	cUTIs, cIAIs, HABP, VABP, and Gram-negative blood stream infections with limited treatment options
2.	Cefepime and enmetazobactam	2024 FDA	β-lactams and BLICs *Cephalosporin and β-lactamase inhibitor*	HABP and cUTIs
3.	Ceftobiprole	2024 FDA	β-lactams and BLICs *Cephalosporin*	ABSSSI, CABP and SAB
4.	Sulbactam and durlobactam	2023 FDA	β-lactams and BLICs *β-lactamase inhibitors*	*A. baumannii–calcoaceticus* complex HABP and VABP in adult patients aged
5.	Cefiderocol	2019 FDA 2020 EMA	β-lactams and BLICs *Cephalosporin*	cUTIs, HABP, VABP, and other serious Gram-negative infections with limited treatment options
6.	Imipenem, cilastatin, and relebactam	2019 FDA 2020 EMA	β-lactams and BLICs *Carbapenem, renal dehydropeptidase inhibitor, and β-lactamase inhibitor*	cUTIs, cIAIs, and HABP/VABP
7.	Meropenem and vaborbactam	2017 FDA 2018 EMA	β-lactams and BLICs *Carbapenem and β-lactamase inhibitor*	cUTIs, cIAIs, HAP/VAP, and bacteremia or other infections caused by CRE with limited treatment options
8.	Pretomanid	2019 FDA 2020 EMA	Nitroimidazole	Pulmonary TB (MDR-TB and RR-TB)
9.	Lefamulin	2019 FDA 2020 EMA	Pleuromutilin	CABP
10.	Omadacycline	2018 FDA	Tetracycline	ABSSSI, CABP
11.	Eravacycline	2018 FDA 2018 EMA	Tetracycline	cIAIs
12.	Plazomicin	2018 FDA	Aminoglycoside	cUTIs
13.	Rifamycin	2018 FDA	Rifamycin	Travelers' diarrhea caused by non-invasive strains of *Escherichia coli*
14.	Delafloxacin	2017 FDA 2019 EMA	Fluoroquinolone	ABSSSIs, CABP
15.	Gepotidacin	2025 FDA	Triazaacenaphthylenes	uUTI

The original version of this article has been updated.

