# Supplementary material for: Molecular resistance mechanisms to newly approved antibiotics (2017–2025) in WHO priority pathogens
Source: Front Microbiol. 2026 Jan 13;16:1719798. doi: 10.3389/fmicb.2025.1719798 (PMC12835396; doi:10.3389/fmicb.2025.1719798)
Supplement: Supplementary file 1 [file Data_Sheet_1.pdf]

Table S1. Reported presence of resistance genes coding for β-lactamases.

Bacterium	β-lactamases	Citation
Acinetobacter baumannii	ADC-57	(1,2)
	CMY-6	(3)
	NDM-1	(1–4)
	NDM-9	(5)
	OXA-23	(1–3,6,7)
	OXA-24	(7)
	OXA-51-like	(2)
	OXA-64	(1)
	OXA-72	(8)
Escherichia coli	CMY-59	(9)
	CMY-145	(10)
	CTX-M-15	(11)
	KPC-3	(11)
	NDM-5	(9–13)
	NDM-35	(14)
	RMTB-1	(11)
	TEM-252	(15)
	VIM-1	(12)
Klebsiella pneumoniae	CTX-M-15	(16–18)
	KPC-2	(19)
	KPC-3	(20)
	KPC-31	(18,21)
	KPC-33	(21,22)
	KPC-62	(18)
	KPC-109	(23)
	KPC-121	(24)

	KPC-203	(25)
	KPC-245	(26)
	NDM-1	(17,20,22,27)
	NDM-5	(16,20)
	NDM-7	(20)
	OXA-48	(28)
	OXA-181	(24,29,30)
	OXA-232	(16,17)
	SHV-12	(31)
Pseudomonas aeruginosa	IMP-8	(32)
	NDM-1	(32,33)
	OXA-2	(34,35)
	OXA-10	(34,35)
	OXA-15	(36)
	OXA-46	(35)
	PER-1	(37,38)
	SHV-12	(36)
	VIM-2	(32)

References

1. Sánchez-Urtaza S, Ocampo-Sosa A, Molins-Bengoetxea A, Rodríguez-Grande J, El-Kholy MA, Hernandez M, et al. Co-Existence of blaNDM-1, blaOXA-23, blaOXA-64, blaPER-7 and blaADC-57 in a Clinical Isolate of Acinetobacter baumannii from Alexandria, Egypt. Int J Mol Sci. 7 agosto 2023;24(15):12515.

2. Sánchez-Urtaza S, Ocampo-Sosa A, Molins-Bengoetxea A, El-Kholy MA, Hernandez M, Abad D, et al. Molecular characterization of multidrug resistant Acinetobacter baumannii clinical isolates from Alexandria, Egypt. Front Cell Infect Microbiol. 2023;13:1208046.

3. Traglia GM, Pasteran F, Moheb S, Akhtar U, Gonzalez S, Maldonado C, et al. Insights into Acinetobacter baumannii AMA205’s Unprecedented Antibiotic Resistance. Int J Mol Sci. 24 ottobre 2024;25(21):11424.

4. Rodríguez-Aguirregabiria M, Lázaro-Perona F, Cacho-Calvo JB, Arellano-Serrano MS, Ramos-Ramos JC, Rubio-Mora E, et al. Challenges Facing Two Outbreaks of Carbapenem-Resistant Acinetobacter baumannii: From Cefiderocol Susceptibility Testing to the Emergence of Cefiderocol-Resistant Mutants. Antibiot Basel Switz. 21 agosto 2024;13(8):784.

5. Gaillot S, Oueslati S, Vuillemenot JB, Bour M, Iorga BI, Triponney P, et al. Genomic characterization of an NDM-9-producing Acinetobacter baumannii clinical isolate and role of Glu152Lys substitution in the enhanced cefiderocol hydrolysis of NDM-9. Front Microbiol. 2023;14:1253160.

6. He Y, Wang Y, Ma X, Zhao L, Guan J, Zhao J, et al. Resistance to Cefiderocol Involved Expression of PER-1 β-Lactamase and Downregulation of Iron Transporter System in Carbapenem-Resistant Acinetobacter baumannii. Infect Drug Resist. 2022;15:7177–87.

7. Desmoulin A, Sababadichetty L, Kamus L, Daniel M, Feletti L, Allou N, et al. Adaptive resistance to cefiderocol in carbapenem-resistant Acinetobacter baumannii (CRAB): Microbiological and clinical issues. Heliyon. 15 maggio 2024;10(9):e30365.

8. Huang E, Thompson RN, Moon SH, Keck JM, Lowry MS, Melero J, et al. Treatment-emergent cefiderocol resistance in carbapenem-resistant Acinetobacter baumannii is associated with insertion sequence ISAba36 in the siderophore receptor pirA. Antimicrob Agents Chemother. 9 luglio 2024;68(7):e0029024.

9. Barker KR, Rebick GW, Fakharuddin K, MacDonald C, Mulvey MR, Mataseje LF. When the Trojan horse is unable to reach inside the city: investigation of the mechanism of resistance behind the first reported cefiderocol-resistant E. coli in Canada. Microbiol Spectr. 2 maggio 2024;12(5):e0322323.

10. Haidar G, Kline EG, Kitsios GD, Wang X, Kwak EJ, Newbrough A, et al. Emergence of high-level aztreonam-avibactam and cefiderocol resistance following treatment of an NDM-producing Escherichia coli bloodstream isolate exhibiting reduced susceptibility to both agents at baseline. JAC-Antimicrob Resist. ottobre 2024;6(5):dlae141.

11. Martin MJ, Luo TL, Kovalchuk V, Kondratiuk V, Dao HD, Kovalenko I, et al. Detection of cefiderocol and aztreonam/avibactam resistance in epidemic Escherichia coli ST-361 carrying blaNDM-5 and blaKPC-3 from foreign fighters evacuated from Ukraine. Antimicrob Agents Chemother. 6 novembre 2024;68(11):e0109024.

12. Kocer K, Boudour-Halil D, Chanthalangsy Q, Sähr A, Heeg K, Boutin S, et al. Genomic Modification of TonB and Emergence of Small-Colony Phenotype in VIM- and NDM-Producing Escherichia coli following Cefiderocol Exposure In Vitro. Antimicrob Agents Chemother. 17 maggio 2023;67(5):e0011823.

13. Wang Q, Jin L, Sun S, Yin Y, Wang R, Chen F, et al. Occurrence of High Levels of Cefiderocol Resistance in Carbapenem-Resistant Escherichia coli before Its Approval in China: a Report from China CRE-Network. Microbiol Spectr. 29 giugno 2022;10(3):e0267021.

14. Poirel L, Ortiz de la Rosa JM, Sakaoglu Z, Kusaksizoglu A, Sadek M, Nordmann P. NDM-35-Producing ST167 Escherichia coli Highly Resistant to β-Lactams Including Cefiderocol. Antimicrob Agents Chemother. 16 agosto 2022;66(8):e0031122.

15. Rodríguez-Villodres Á, Ortiz de la Rosa JM, Gálvez-Benítez L, Gascón ML, Peñalva G, Dorado Pardo FJ, et al. Survival of infection with TEM β-lactamase-producing Escherichia coli with Pan-β-lactam resistance. J Infect. novembre 2024;89(5):106268.

16. Ranieri SC, Fabbrizi V, D' Amario AM, Frascella MG, Di Biase V, Di Francesco C, et al. First report of a bla NDM-producing extensively drug resistant Klebsiella pneumoniae ST437 in Italy. Front Cell Infect Microbiol. 2024;14:1426817.

17. Daoud L, Allam M, Collyns T, Ghazawi A, Saleem A, Al-Marzooq F. Extreme resistance to the novel siderophore-cephalosporin cefiderocol in an extensively drug-resistant Klebsiella pneumoniae strain causing fatal pneumonia with sepsis: genomic analysis and synergistic combinations for resistance reversal. Eur J Clin Microbiol Infect Dis Off Publ Eur Soc Clin Microbiol. novembre 2023;42(11):1395–400.

18. Castillo-Polo JA, Hernández-García M, Morosini MI, Pérez-Viso B, Soriano C, De Pablo R, et al. Outbreak by KPC-62-producing ST307 Klebsiella pneumoniae isolates resistant to ceftazidime/avibactam and cefiderocol in a university hospital in Madrid, Spain. J Antimicrob Chemother. 3 maggio 2023;78(5):1259–64.

19. Zhou Y, Wu X, Wu C, Zhou P, Yang Y, Wang B, et al. Emergence of KPC-2 and NDM-5-coproducing hypervirulent carbapenem-resistant Klebsiella pneumoniae with high-risk sequence types ST11 and ST15. mSphere. 30 gennaio 2024;9(1):e0061223.

20. Long H, Feng Y, Zong Z. Global molecular epidemiology of the incomplete CirA protein related to cefiderocol resistance in Klebsiella pneumoniae: a genome-based study. Microbiol Spectr. 6 maggio 2025;13(5):e0141024.

21. Birgy A, Nnabuife C, Palzkill T. The mechanism of ceftazidime and cefiderocol hydrolysis by D179Y variants of KPC carbapenemases is similar and involves the formation of a long-lived covalent intermediate. Antimicrob Agents Chemother. 6 marzo 2024;68(3):e0110823.

22. Bellinzona G, Merla C, Corbella M, Iskandar EN, Seminari E, Di Matteo A, et al. Concomitant Resistance to Cefiderocol and Ceftazidime/Avibactam in Two Carbapenemase-Producing Klebsiella pneumoniae Isolates from Two Lung Transplant Patients. Microb Drug Resist Larchmt N. gennaio 2024;30(1):21–6.

23. Di Pilato V, Codda G, Niccolai C, Willison E, Wong JLC, Coppo E, et al. Functional features of KPC-109, a novel 270-loop KPC-3 mutant mediating resistance to avibactam-based β-lactamase inhibitor combinations and cefiderocol. Int J Antimicrob Agents. gennaio 2024;63(1):107030.

24. Gaibani P, Amadesi S, Lazzarotto T, Ambretti S. Genome characterization of a Klebsiella pneumoniae co-producing OXA-181 and KPC-121 resistant to ceftazidime/avibactam, meropenem/vaborbactam, imipenem/relebactam and cefiderocol isolated from a critically ill patient. J Glob Antimicrob Resist. settembre 2022;30:262–4.

25. Amadesi S, Bianco G, Secci B, Fasciana T, Boattini M, Costa C, et al. Complete Genome Sequence of a Klebsiella pneumoniae Strain Carrying Novel Variant blaKPC-203, Cross-Resistant to Ceftazidime/Avibactam and Cefiderocol, but Susceptible to Carbapenems, Isolated in Italy, 2023. Pathog Basel Switz. 15 giugno 2024;13(6):507.

26. Vaiana C, Vazzana R, Castelbuono S, Cona A, Mularoni A, Minucci R, et al. Whole-genome sequencing characterisation of a new KPC-245 variant-carrying Klebsiella pneumoniae strain isolated from a transplanted patient and resistant to ceftazidime/avibactam, meropenem/vaborbactam and imipenem/relebactam. J Glob Antimicrob Resist. maggio 2025;42:229–33.

27. Coppi M, Antonelli A, Niccolai C, Bartolini A, Bartolini L, Grazzini M, et al. Nosocomial outbreak by NDM-1-producing Klebsiella pneumoniae highly resistant to cefiderocol, Florence, Italy, August 2021 to June 2022. Euro Surveill Bull Eur Sur Mal Transm Eur Commun Dis Bull. ottobre 2022;27(43):2200795.

28. Yang C, Wang L, Lv J, Wen Y, Gao Q, Qian F, et al. Effects of different carbapenemase and siderophore production on cefiderocol susceptibility in Klebsiella pneumoniae. Antimicrob Agents Chemother. 5 dicembre 2024;68(12):e0101924.

29. Bovo F, Amadesi S, Palombo M, Lazzarotto T, Ambretti S, Gaibani P. Clonal dissemination of Klebsiella pneumoniae resistant to cefiderocol, ceftazidime/avibactam, meropenem/vaborbactam and imipenem/relebactam co-producing KPC and OXA-181 carbapenemase. JAC-Antimicrob Resist. agosto 2023;5(4):dlad099.

30. Gaibani P, Amadesi S, Lazzarotto T, Ambretti S. Complete Genome Sequence of a Multidrug-Resistant Klebsiella pneumoniae Strain Carrying blaOXA181 and blaKPC-125 Carbapenemase. Microb Drug Resist Larchmt N. settembre 2022;28(9):916–20.

31. Liu C, Yi J, Lu M, Yang P, Du C, Jiang F, et al. Dynamic within-host cefiderocol heteroresistance caused by blaSHV-12 amplification in pandrug-resistant and hypervirulent Klebsiella pneumoniae sequence type 11. Drug Resist Updat Rev Comment Antimicrob Anticancer Chemother. marzo 2024;73:101038.

32. Benzaarate I, El Otmani F, Khazaz A, Timinouni M, Bourjilat F, Bogaerts P, et al. Detection of Carbapenemase Encoding Gene and Resistance to Cefiderocol in Hospital and Community eXtensive Drug Resistance and Carbapenem-Resistant Pseudomonas aeruginosa Strains in Morocco. Foodborne Pathog Dis. ottobre 2023;20(10):460–6.

33. González-Pinto L, Alonso-García I, Blanco-Martín T, Camacho-Zamora P, Fraile-Ribot PA, Outeda-García M, et al. Impact of chromosomally encoded resistance mechanisms and transferable β-lactamases on the activity of cefiderocol and innovative β-lactam/β-lactamase inhibitor combinations against Pseudomonas aeruginosa. J Antimicrob Chemother. 1 ottobre 2024;79(10):2591–7.

34. Oliver A, Arca-Suárez J, Gomis-Font MA, González-Pinto L, López-Causapé C. Emerging resistance mechanisms to newer β-lactams in Pseudomonas aeruginosa. Clin Microbiol Infect Off Publ Eur Soc Clin Microbiol Infect Dis. 20 marzo 2025;S1198-743X(25)00131-4.

35. Vuillemin X, Da Silva M, Bour M, Landon C, Plésiat P, Jeannot K. Cefiderocol activity is compromised by acquired extended-spectrum oxacillinases in Pseudomonas aeruginosa. Int J Antimicrob Agents. settembre 2023;62(3):106917.

36. González-Pinto L, Blanco-Martín T, Alonso-García I, Rodríguez-Pallares S, Outeda-García M, Gomis-Font MA, et al. Impact of transferable β-lactamases and intrinsic AmpC amino acid substitutions on the activity of cefiderocol against wild-type and iron uptake-deficient mutants of Pseudomonas aeruginosa. J Antimicrob Chemother. 4 novembre 2024;79(11):3023–8.

37. Papa-Ezdra R, Cordeiro NF, Outeda M, Garcia-Fulgueiras V, Araújo L, Seija V, et al. Novel Resistance Regions Carrying TnaphA6, blaVIM-2, and blaPER-1, Embedded in an ISPa40-Derived Transposon from Two Multi-Resistant Pseudomonas aeruginosa Clinical Isolates. Antibiot Basel Switz. 2 febbraio 2023;12(2):304.

38. Wang L, Zhang X, Zhou X, Bi Y, Wang M, Guo Q, et al. Insertion of ISPa1635 in ISCR1 Creates a Hybrid Promoter for blaPER-1 Resulting in Resistance to Novel β-lactam/β-lactamase Inhibitor Combinations and Cefiderocol. Antimicrob Agents Chemother. 15 giugno 2023;67(6):e0013523.
